# Scientific publications from Arab world in leading journals of Integrative and Complementary Medicine: a bibliometric analysis

**DOI:** 10.1186/s12906-015-0840-z

**Published:** 2015-09-04

**Authors:** Sa’ed H. Zyoud, Samah W. Al-Jabi, Waleed M. Sweileh

**Affiliations:** Poison Control and Drug Information Center (PCDIC), College of Medicine and Health Sciences, An-Najah National University, Nablus, Palestine; Department of Clinical and Community Pharmacy, College of Medicine and Health Sciences, An-Najah National University, Nablus, Palestine; WHO Collaborating Centre for Drug Information, National Poison Centre, Universiti Sains Malaysia (USM), Penang, Malaysia; Department of Pharmacology and Toxicology, College of Medicine and Health Sciences, An-Najah National University, Nablus, Palestine

**Keywords:** Bibliometric, Integrative and Complementary Medicine, Arab world, Web of Science, *h*-index

## Abstract

**Background:**

Bibliometric analysis is increasingly employed as a useful tool to assess the quantity and quality of research performance. The specific goal of the current study was to evaluate the performance of research output originating from Arab world and published in international Integrative and Complementary Medicine (ICM) journals.

**Methods:**

Original scientific publications and reviews from the 22 Arab countries that were published in 22 international peer-reviewed ICM journals during all previous years up to December 31^st^ 2013, were screened using the Web of Science databases.

**Results:**

Five hundred and ninety-one documents were retrieved from 19 ICM journals. The *h*-index of the set of papers under study was 47. The highest *h*-index was 27 for Morocco, 21 for Jordan, followed by 19 for each Kingdom of Saudi Arabia (KSA), and Egypt, and the lowest *h*-index was 1 for each of Comoros, Qatar, and Syrian Arab Republic. No data related to ICM were published from Djibouti, and Mauritania. After adjusting for economy and population power, Somalia (89), Morocco (32.5), Egypt (31.1), Yemen (21.4), and Palestine (21.2) had the highest research productivity. The total number of citations was 9,466, with an average citation of 16 per document. The study identified 262 (44.3 %) documents with 39 countries in Arab-foreign country collaborations. Arab authors collaborated most with countries in Europe (24.2 %), followed by countries in the Asia-Pacific region (9.8 %).

**Conclusion:**

Scientific research output in the ICM field in the Arab world region is increasing. Most of publications from Arab world in ICM filed were driven by societal use of medicinal plants and herbs. Search for new therapies from available low cost medicinal plants in Arab world has motivated many researchers in academia and pharmaceutical industry. Further investigation is required to support these findings in a wider journal as well as to improve research output in the field of ICM from Arab world region by investing in more national and international collaborative research project.

## Background

Recent research has shown that Integrative and Complementary Medicine (ICM) becomes increasingly popular and commonly used by the general population [1]. ICM is a growing scientific field and during the last decades, there has been a rapid rising of peer-reviewed ICM publications in a variety of scientific journals [1–4]. ICM is recognized as a relative expression including a variety of health-care methods, which are known “other than” conventional medicine [5]. Previously, the concept “complementary and alternative medicine” (CAM) has been commonly used, along with transformations of western health care, in various contexts; but it has been recently substituted by the concept of “integrated and complementary medicine”. In general, “complementary medicine” intended to treatments that are used together with conventional medicine, while “alternative medicine” intended to treatments that are used in place of conventional medicine [2, 6]. Integrative medicine is considered as an integration between the alternative medicine practices and methods with conventional medicine [6, 7].

During the last decade, several researchers had analyzed and assessed the outcome of scientific research production from Arab world [8–15]. Actually, the assessment of scientific research production in the ICM field has been insufficiently investigated to date, and there are a small number of worldwide published studies on scientific research production in ICM [1–3, 5, 16, 17]. On the other hand, within the limits of our knowledge, there is no previous report regarding the assessment of scientific research production in ICM originating from the Arab region. However, the status of ICM research in this region, until now, has not been reported. Thus, estimation of Arab output of current research in ICM may be of attention. Therefore, in this type of bibliometric study, we wanted to assess the quantity and quality [18] of published papers in the ICM field which was published in the world’s leading ICM journals from the Arab region.

## Methods

### Search strategy

Scientific research output in the ICM field was evaluated based on an indicators which are commonly used in previous similar bibliometric studies [1–3, 5, 15–17, 19–24]. The information used in this study was extracted from the Science Citation Index (SCI). The source of journals for the publications examined was chosen with the intention to represent Arab world scientific research output in the field of ICM. Twenty-two journals were included in “Integrative & Complementary Medicine” category in the Journal Citation Report (JCR) – 2013. It looks that publications published in these 22 journals represent the majority of scientific research output in ICM field, even though there are definitely other ICM publications may be published in other fields.

All Arab countries: Jordan; Iraq; Syrian Arab Republic (SAR); Kuwait; Egypt; Yemen; Qatar; United Arab Emirates (UAE); Bahrain; Kingdom of Saudi Arabia (KSA); Oman; Sudan; Tunisia; Algeria; Lebanon; Libya; Morocco; Somalia; Djibouti; Comoros; and Mauritania were used as country keys followed by “Integrative & Complementary Medicine” phrase as ISI Web of Knowledge's category. Palestine was excluded from the search keys and replaced by Israel in separate search because the ISI Web of Knowledge database does not identify Palestine as an independent country yet, and then we refined the organizations only to those related to Palestine.

To increase the precision of results, original scientific publications and reviews from the 22 Arab countries that were published in 22 international peer-reviewed ICM journals during all previous years up to December 31^st^ 2013, were screened because it seems that this type of publications represent and describe the research activities. Other types of publications such as letter to the editor, editorials, and others were excluded. All searches and data extraction were accomplished within one day on 1^st^ August 2014 to avoid the possibility of unfairness due to the daily update of databases.

### Statistical analysis

The extracted data from ISI Web of Knowledge were transferred to Microsoft Office Excel® 2007. The data were then exported to the Statistical Package for the Social Sciences (SPSS) version 15 for analysis. Numerical data such as total number of citations are presented as average and categorical data are presented as frequencies and percentages. Multidimensional scaling (MDS) using PROXSCAL analysis with Euclidean distances was used to visualize and verify countries' collaboration profiles in a graphical way [25, 26]. The extracted data were used to create the following information: (a) total and trends of Arab world contributions in ICM research; (b) Arab countries scientific research output and collaboration patterns in the field of ICM; (c) ICM journals with their impact factors according to Institute for Scientific Information (ISI) journal citation reports (JCR) 2012 in which Arab authors published their work; and (d) the citations pattern. Only the 10 top ranked measurements (e.g. cited articles, countries, institutions) were taken into consideration, and converted to rank order by the standard competition ranking (SCR) [20]. The *h*-index for data collected from SCI and for each country is presented as a way of qualifying research performance, which was recognized by Hirsch in 2005, where index *h* is known as the number of publication with a citation number more than or equal to *h* [27].

## Results

**Fig. 1:**
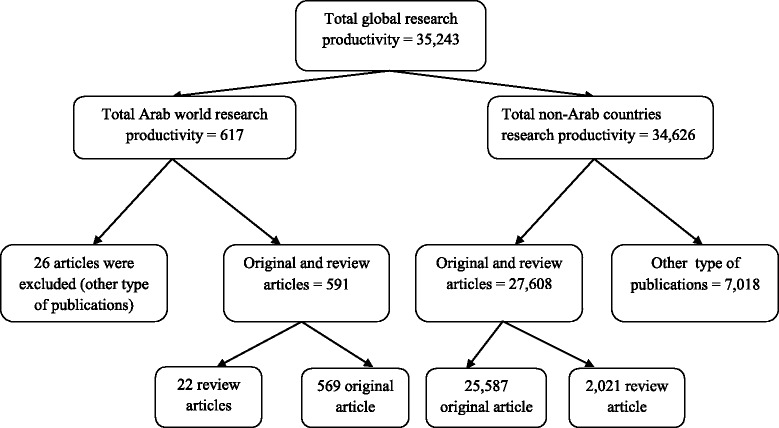
Diagrammatic representation of global research productivity profiles in leading journals of integrative and complementary medicine

The total number of documents related to ICM obtained by using the key words “Integrative & Complementary Medicine” in SCI search engine as Web of Science Category without specifying the name of any country and by using the same inclusion criteria was 28,199 documents (Fig. 1). This number of publications represents the total global research output in ICM field. By using the same methodology, only 591 (2.1 % from the total global research output in ICM) documents were from the 22 countries retrieved; comprising 569 (96.3 %) original journal articles, and 22 (3.7 %) review articles (Fig. 1). The annual number of documents published in the period of publication (1980–2013) indicated that research activity from Arab world was low in the earliest two decades but demonstrated a noticeable increase in the last decade (Table 1). Furthermore, Arab research output during the recent decade was low in the first years but demonstrated a noticeable increase after 2011. Around 34.2 % of publications were published during 2011–2013; however, the total number of scientific research output in 2013 may be a little bit rising because it may still open for new journals issues. The first article published from Arab World was in Egypt, and it was published by Sayed MD in *Journal of Ethnopharmacology* in 1980 [28].

**Table 1:** Annual number of integrative & complementary medicine-based publications from Arab World

Year	Total N = 591 (%)
1980-1989	29 (4.9)
1990-1999	82 (13.9)
2000	25 (4.2)
2001	20 (3.4)
2002	22 (3.7)
2003	23 (3.9)
2004	24 (4.1)
2005	24 (4.1)
2006	20 (3.4)
2007	20 (3.4)
2008	27 (4.6)
2009	33 (5.6)
2010	40 (6.8)
2011	53 (9.0)
2012	74 (12.5)
2013	66 (12.7)

The extracted publications were published in 19 out of 22 peer-reviewed ICM journals listed in Web of Knowledge® (Table 2). Three hundred and twenty-five articles (55.0 %) were published in *Journal of Ethnopharmacology*, whereas 64 (10.8 %) were published in *Evidence Based Complementary and Alternative Medicine*, and 53 (9.0 %) were published in *Phytomedicine*. This was followed by 51 (8.6 %) articles that were published in *BMC Complementary and Alternative Medicine,* and 28 (4.7 %) were published in *American Journal of Chinese Medicine.* Of the 19 journal titles, 17 (89.5 %) have their IF listed in the JCR 2012 (Table 2).

**Table 2:** List of journals in which the 591 documents were published with their impact factors

Journal	Frequency (%)	IF^a^
*Journal of Ethnopharmacology*	325 (55.0)	2.755
*Evidence Based Complementary and Alternative Medicine*	64 (10.8)	1.722
*Phytomedicine*	53 (9.0)	2.972
*BMC Complementary And Alternative Medicine*	51 (8.6)	2.082
*American Journal of Chinese Medicine*	28 (4.7)	2.281
*African Journal of Traditional Complementary and Alternative Medicines*	26 (4.4)	0.518
*Journal of Alternative and Complementary Medicine*	14 (2.4)	1.464
*Complementary Therapies in Medicine*	7 (1.2)	2.093
*Integrative Cancer Therapies*	5 (0.9)	2.354
*Journal of Manipulative and Physiological Therapeutics*	4 (0.7)	1.647
*Journal of Herbal Medicine*	2 (0.3)	NA
*Homeopathy*	2 (0.3)	0.838
*Forschende Komplementarmedizin*	2 (0.3)	1.279
*European Journal of Integrative Medicine*	2 (0.3)	0.559
*Chinese Journal of Natural Medicines*	2 (0.3)	1.059
*Journal of Traditional Chinese Medicine*	1 (0.2)	0.589
*Holistic Nursing Practice*	1 (0.2)	0.341
*Chinese Medicine*	1 (0.2)	NA
*Alternative Medicine Review*	1 (0.2)	4.857

*Abbreviations: IF* impact factor, *NA* not available

^a^The impact factor was reported according to Institute for Scientific Information (ISI) journal citation reports (JCR) 2012

The highest number of publications in ICM journals was from KSA (25.0 %), followed by Egypt (16.8), and Morocco (16.2 %); (Table 3). No data related to ICM were published from Djibouti, and Mauritania. After adjusting for economy and population power, Somalia (89), Morocco (32.5), Egypt (31.1), Yemen (21.4), and Palestine (21.2) had the highest research output. Rank of countries such as Qatar, and Kuwait tended to be comparatively low. The total number of citations, at the date of data extraction, was 9,466, with an average citation of 16.0 per document. The highest average number of citations was 29 for Libya, followed by 25.4 for Palestine, and the lowest average number of citations was 2.8 for Bahrain and 6.7 for Tunisia. The total number of citations without self-citation was 8,842. Of the 591 documents used for calculation of *h*-index, 47 documents had been cited at least 47 times. The highest *h*-index was 27 for Morocco, and 21 for each Jordan and Egypt, followed by 19 for KSA, and the lowest *h*-index was 1 for Comoros, Qatar, and SAR. Additionally, the highest country collaborated with international authors was achieved by the KSA, with 70 documents, followed by 64 documents for Morocco and 46 documents for Egypt.

**Table 3:** Bibliometric analysis of the 591 documents by country

SCR^a^	Countries	No. of articles (%)	*h*-index	Average of citation	No. of foreign countries that the main country collaborated with	No. of documents with international collaborations	Adjustment index^b^
1^st^	KSA	148 (25.0)	19	9.2	24	70	5.89
2^nd^	Egypt	99 (16.8)	21	13.3	27	46	31.06
3^nd^	Morocco	96 (16.2)	27	24.5	16	64	32.53
4^th^	Jordan	52 (8.8)	21	22.1	8	9	10.52
5^th^	Tunisia	33 (5.6)	10	6.7	7	14	7.79
6^th^	Yemen	32 (5.4)	12	15.1	12	26	21.41
7^th^	Algeria	24 (4.1)	9	11.2	9	14	4.49
8^th^	Palestine	21 (3.6)	11	28.1	4	5	21.16
8^th^	Iraq	21 (3.6)	11	22.4	4	7	3.25
10^th^	Lebanon	20 (3.4)	10	14.2	7	10	2.06
11^th^	Sudan	19 (3.2)	10	17.8	11	12	12.03
12^th^	UAE	18 (3.1)	10	17.6	10	8	0.48
12^th^	Oman	18 (3.1)	7	13.5	14	11	0.85
14^th^	Somalia	8 (1.4)	6	17.1	2	6	88.99
15^th^	Kuwait	5 (0.9)	4	11.6	0	0	0.1
16^th^	Bahrain	4 (0.7)	2	2.8	4	4	0.18
17^th^	Libya	3 (0.5)	3	29	3	2	0.3
18^th^	SAR	1 (0.2)	1	-	2	1	0.3
18^th^	Qatar	1 (0.2)	1	-	1	1	0.01
18^th^	Comoros	1(0.2)	1	-	1	1	1.2
21^st^	Djibouti	0 (0.0)	-	-	-	-	-
21^st^	Mauritania	0 (0.0)	-	-	-	-	-

*Abbreviations: KSA* Kingdom of Saudi Arabia, *SAR* Syrian Arab Republic, *SCR* Standard Competition Ranking, *UAE* United Arab Emirates

^a^Equal countries have the same ranking number, and then a gap is left in the ranking numbers

^b^An adjustment index (AI) was calculated using the following formula: AI = [total number of publications for the country / GDP per capita of the country]*1000. Where: GDP per capita = GDP/population of the country

**Fig. 2:**
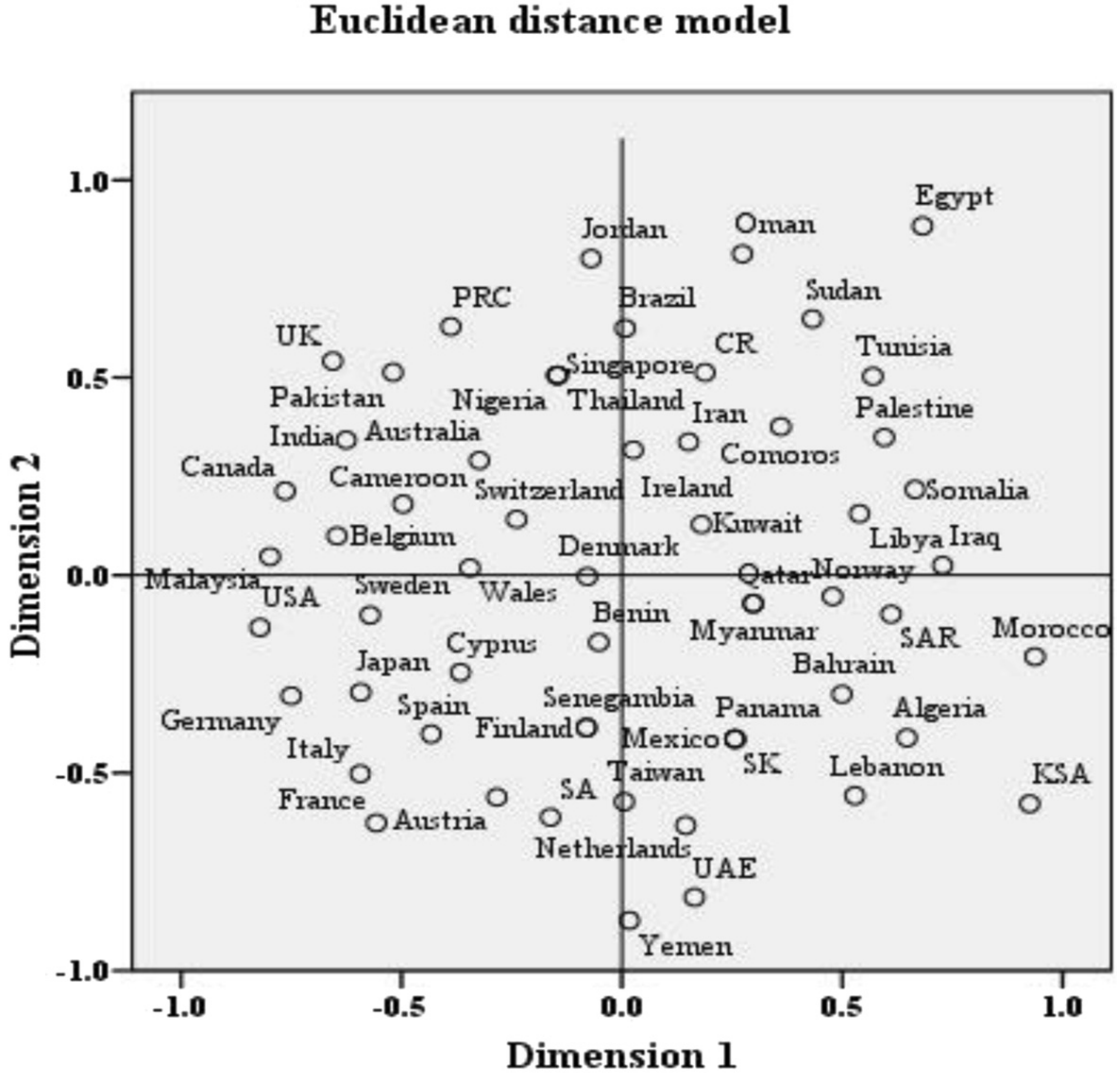
Multidimensional scaling (MDS) for collaboration profile for Arab countries using Euclidean distance model

In addition, the study identified 262 (44.3 %) documents with 39 countries in Arab-foreign country collaborations. Arab authors actively worked in partnership from France (n = 48), followed by Germany (n = 34), Malaysia (n = 30), and the United States of America (USA); (n = 25); (Table 4). By region, Arab authors actively worked in partnership from countries in Europe (24.2 %), particularly France and Germany, followed by countries in the Asia-Pacific region (9.8 %), particularly India and Pakistan (Table 4). Figure 2 illustrates the multidimensional scaling map of the collaborations correlation matrix of 59 countries over the study period. Mapping the data along two dimensions allows us to visualize the correspondence between documents in terms of their relative distances based on the collaborations profiles with certain country. The more closely the two countries were represented on the map, the more frequently they were collaborated jointly by the 262 collaborated documents. Collaboration map with statistically obtained values for configuration were derived along two dimensions. S-stress is a measure of fit ranging from 0 (perfect fit) to 1 (worst possible fit). Stress measure for the results of the current study was 0.3 (good fit) while the squared correlation (RSQ) was 0.91 meaning that 91 % of variance in the model could be explained by the two dimensions [25, 26]. In this case, we have a good fit model representing a poor approximation of the Arab-foreign country collaboration. Morocco and KSA were strong on dimension 1 while Egypt appeared high on dimension 2. Referring to stimulus coordinates (i.e. Common Space), we observe that values range from −0.82 to 0.94 for dimension 1 and from −0.87 to 0.88 for dimension 2.

**Table 4:** Collaborations between Arab countries and foreign countries in Integrative & Complementary Medicine publications

Collaborating countries	No. of documents (%)	Collaborating countries	No. of documents (%)
Arab-Europe	143 (24.2)	Arab-Americas	39 (6.6)
France	48 (8.1)	USA	25 (4.2)
Germany	34 (5.8)	Canada	12 (2.0)
Sweden	13 (2.2)	Brazil	2 (0.3)
Italy	12 (2.0)	Mexico	1 (0.2)
UK	11 (1.9)	Panama	1 (0.2)
Spain	10 (1.7)	Arab-Other Middle East, Africa	16 (2.7)
Belgium	8 (1.4)	Cameroon	5 (0.9)
Switzerland	4 (0.7)	South Africa	4 (0.7)
Wales	3 (0.5)	Iran	4 (0.7)
Austria	2 (0.3)	Benin	1 (0.2)
Netherlands	2 (0.3)	Nigeria	1 (0.2)
Denmark	2 (0.3)	Senegambia	1 (0.2)
Czech Republic	1 (0.2)	Arab-Asia-Pacific	58 (9.8)
Finland	1 (0.2)	India	19 (3.2)
Ireland	1 (0.2)	Pakistan	12 (2.0)
Cyprus	1 (0.2)	Japan	12 (2.0)
Norway	1 (0.2)	PRC	8 (1.4)
Arab-Southeast Asia	33 (5.9)	Australia	5 (0.9)
Malaysia	30 (5.1)	Taiwan	2 (0.3)
Singapore	1 (0.2)	South Korea	1 (0.2)
Thailand	1 (0.2)	Arab-Arab	33 (5.9)
Myanmar	1 (0.2)		

*Abbreviations: PRC* People's Republic of China, *UK* United Kingdom, *USA* United States of America

Table 5 shows the areas of interest of the scientific articles. Category of pharmacology and pharmacy was the most areas of research interest, represented by 382 (64.6 %) articles. The second most researched topic was plant sciences 378 (63.9 %) followed by general internal medicine 28 (4.7 %). Table 6 shows a list of the 20 most cited articles in ICM field originating from Arab region. Table 7 shows the top 20 most prolific institutions in ICM journals. The most prolific institution was *King Saud University* (15.4 %), followed by *University of Jordan* (6.1 %), and *Cairo University* (4.4 %).

**Table 5:** Areas of interest for published papers by the Arab countries

Areas of interest	n (%)^a^
Pharmacology and pharmacy	382 (64.6)
Plant sciences	378 (63.9)
General internal medicine	28 (4.7)
Oncology	5 (0.9)
Health care sciences services	4 (0.7)
Rehabilitation	4 (0.7)
Nursing	1 (0.2)

^a^Total exceeds 100 % as data are overlapping due to multidiscipline interaction

**Table 6:** Ranking of top 20 cited Integrative & Complementary Medicine articles from Arab world

SCR^a^	Title	Authors-Year	Source title	Cited by
1^st^	Phytotherapy of hypertension and diabetes in oriental Morocco	Ziyyat et al. 1997	*Journal of Ethnopharmacology*	148
2^nd^	Screening of some Palestinian medicinal plants for antibacterial activity	Essawi and srour 2000	*Journal of Ethnopharmacology*	144
3^rd^	Hypoglycaemic effect of Artemisia herba alba. II. Effect of a valuable extract on some blood parameters in diabetic animals.	Al-Shamaony et al. 1994	*Journal of Ethnopharmacology*	139
4^th^	Studies on the antimicrobial activity of Nigella sativa seed (black cumin)	Hanafy and Hatem 1991	*Journal of Ethnopharmacology*	116
5^th^	The anti-inflammatory, analgesic and antipyretic activity of Nigella sativa	Al-Ghamdi 2001	*Journal of Ethnopharmacology*	113
6^th^	Ethnopharmacological survey of medicinal plants used for the treatment of diabetes mellitus, hypertension and cardiac disease in the south-east region of Morocco (Tafilalet)	Eddouks et al. 2002	*Journal of Ethnopharmacology*	101
7^th^	Ethnobotanical survey in the Palestinian area: a classification of the healing potential of medicinal plants	Ali-Shtayeh et al. 2000	*Journal of Ethnopharmacology*	98
7^th^	Anti-nociceptive and anti-inflammatory effects of some Jordanian medicinal plant extracts	Atta and Alkofahi 1998	*Journal of Ethnopharmacology*	98
7^th^	Ethnobotanical survey of medicinal plants used for the treatment of diabetes, cardiac and renal diseases in the North centre region of Morocco (Fez-Boulemane)	Jouad et al. 2001	*Journal of Ethnopharmacology*	98
10^th^	Repertory of standard herbal drugs in the Moroccan pharmacopoea.	Bellakhdar et al. 1991	*Journal of Ethnopharmacology*	94
11^th^	Antimicrobial activity of 20 plants used in folkloric medicine in the Palestinian area	Ali-Shtayeh et al. 1998	*Journal of Ethnopharmacology*	93
12^th^	Ethnopharmacological survey of plants used in the traditional treatment of hypertension and diabetes in south-eastern Morocco (Errachidia province)	Tahraoui et al. 2007	*Journal of Ethnopharmacology*	91
12^th^	Chemical composition, antibacterial and antifungal activities of the essential oil of Haplophyllum tuberculatum from Oman	Al-Burtamani et al. 2005	*Journal of Ethnopharmacology*	91
12^th^	Screening of Yemeni medicinal plants for antibacterial and cytotoxic activities	Ali et al. 2001	*Journal of Ethnopharmacology*	91
15^th^	Potential antimalarial candidates from African plants: and in vitro approach using Plasmodium falciparum	Khalid et al. 1986	*Journal of Ethnopharmacology*	88
16^th^	Evaluation of mastic, a crude drug obtained from Pistacia lentiscus for gastric and duodenal anti-ulcer activity.	Alsaid et al. 1986	*Journal of Ethnopharmacology*	84
17^th^	Thymoquinone attenuates ifosfamide-induced Fanconi syndrome in rats and enhances its antitumor activity in mice	Badary 1999	*Journal of Ethnopharmacology*	78
18^th^	Chemical composition and antifungal activity of essential oils of seven Moroccan Labiatae against Botrytis cinerea Pers : Fr.	Bouchra et al. 2003	*Journal of Ethnopharmacology*	77
19^th^	Screening of selected indigenous plants of Lebanon for antimicrobial activity	Barbour et al. 2004	*Journal of Ethnopharmacology*	69
20^th^	The effect of Nigella sativa oil against the liver damage induced by Schistosoma mansoni infection in mice	Mahmoud et al. 2002	*Journal of Ethnopharmacology*	69

*Abbreviation: SCR* Standard Competition Ranking

^a^Equal articles have the same ranking number, and then a gap is left in the ranking numbers

**Table 7:** Ranking of top 20 productive institutions from Arab world affiliations during the study period

SCR^a^	Institutions, country	No. of documents (%)
1^st^	King Saud University, KSA	91 (15.4)
2^nd^	The University of Jordan, Jordan	36 (6.1)
3^rd^	Cairo University, Egypt	26 (4.4)
4^th^	National Research Center, Egypt	25 (4.2)
5^th^	Sanaa University, Yemen	23 (3.9)
6^th^	King Abdulaziz University, KSA	18 (3.1)
7^th^	Université Sidi Mohamed Ben Abdellah, Morocco	13 (2.23)
7^th^	American University of Beirut, Lebanon	13 (2.2)
9^th^	Arab American University, Palestine	12 (2.0)
9^th^	University of Khartoum, Sudan	12 (2.0)
11^th^	Sultan Qaboos University, Oman	11 (1.9)
12^th^	Jazan University, KSA	10 (1.7)
12^th^	Cadi Ayyad University, Morocco	10 (1.7)
12^th^	Al-Azhar University, Egypt.	10 (1.7)
15^th^	King Faisal University, KSA	9 (1.5)
15^th^	Jordan University of Science and Technology, Jordan	9 (1.5)
15^th^	Alexandria University, Egypt	9 (1.5)
15^th^	Ain Shams University, Egypt	9 (1.5)
19^th^	Université de Monastir, Tunisia	8 (1.4)
19^th^	UFR PNPE, Morocco	6 (1.0)
19^th^	Somali National University, Somali	6 (1.0)
19^th^	Scientific Research Council, Iraq	6 (1.0)
19^th^	Mansoura University, Egypt	6 (1.0)
19^th^	An-Najah National University, Palestine	6 (1.0)

*Abbreviations: KSA* Kingdom of Saudi Arabia, *SCR* Standard Competition Ranking, *USA* United States of America

^a^Equal institutes have the same ranking number, and then a gap is left in the ranking numbers

## Discussion

This study was restricted to 591 documents retrieved from ISI Web of Knowledge, bearing Arab countries affiliation and, thus, cannot be generalized to the ICM research activity obtained by other sources such as Google Scholar or Scopus. To our knowledge, this is the first report to analyze the quality and quantity of ICM-based research from the Arab region. bibliometric indicators demonstrated that research output in the field of ICM was low in most Arab countries. The lack of industry-academia partnership in applied health research (including government-academia partnerships), and a general weakness in scientific writing may lead to the lack of scientific research in most Arab regions [14, 29–31]. The total number of ICM documents found in ISI Web of Knowledge between 1980 and 2013 demonstrated a yearly increase. The annual number of documents published in the period of publication (1980–2013) indicated that research production from Arab World was low in the earliest two decades but showed a noticeable increase in the recent decade. A possible explanation for this change in publication output could be attributed to an increase in the number of specialized ICM journals indexed in ISI [1]. On the other hand, ICM research output has pursued the general growth in scientific research output observed in the recent decade and mainly in recent years [1–3, 5, 16, 17]. Furthermore, ICM research productivity has followed the biomedical research output in the Arab region especially in the recent decade [8, 11, 29, 30].

In the current study, the research output for each country was different in comparing to others. Our findings demonstrated that there were a few countries, such as KSA, Morocco and, Egypt where their total ICM research output was obviously superior to that in the other remaining Arab countries. Earlier studies showed that KSA and Egypt or Morocco had the highest research productivity among the Arab countries [23, 29, 32]. On the other hand, after adjusting for economic growth and population size, Somalia, Morocco, Palestine, Egypt, and Yemen demonstrated the highest research output. We didn’t find any report concerning ICM in Arab world, thus we are incapable of interpreting this result in the light of other results. On the other hand, some precious studies using the same bibliometric instrument for research assessment have demonstrated similar result [8, 14, 15, 29, 30, 33, 34]. Countries with fast growing economies such as KSA had higher number of ICM research output. Furthermore, population size is another factor related to enhance research output in the field of ICM such as in Egypt. Our results confirmed that research activity depended on economic growth, population size, or overall scientific research output of each country [15, 35].

In the current study, the average citation rate for ICM publications originating from Arab region was 16 citations per document. This result was slightly higher than the average citations of ICM journals [1–3, 5, 16, 17]. Overall, ICM journals have higher citation numbers in comparing to other categories in ISI Web of Knowledge [23, 36]. This is likely related to several factors. First, even if it is reasonable to argue that ICM is a growing scientific field, it is a highly competitive area in terms of scientific legitimacy [2]. Second, a great majority of the publications in our study are original research articles, and this is supported by previous studies that found that the most cited documents are reviews and original studies [2, 3, 5]. Third, the establishment of ICM as a scientific field is also manifested in increasing numbers of scientific forums that may improve opportunities to get more citations or research funds [2]. In our study, research activity related to ICM started in the 1980s and obviously increased after 2000, while the bulk of the publications (34.2 %) were published from 2011 to 2013. The publications that were published before 2000 were most frequently cited articles and there was a relationship between the number of citations and started publications year [3]. A previous study showed that highly cited publications are usually involving international collaboration and often authored by a large number of scientists [37]. The citation is used as a key indicator of research quality in the study. Highly cited publications positively correlated to the *h-*index of the institution and country individual author, and to individual author [38–41]. Furthermore, *h*-index for ICM publications from Arab countries was 47. This finding was slightly higher than other scientific disciplines [36, 42], and slightly lower once compared to another scientific discipline from Arab countries [23]. One probable explanation for variations in average total citation and *h*-index between ICM publications of Arab countries and those for other non- ICM publications is the IF of ICM journals in which authors published their works. It is believed that articles published in high IF journals have higher possibility of being cited, whereas this is not definite [15]. The *h-*index is a simple way of measuring performance, impact, visibility, and quality of research. Newly published documents have a clear disadvantage because of the short publication period and they should not be compared with documents with longer publication period. Another problem is how to deal with multi-authored papers. Furthermore, in citation analysis there is a problem of how to treat self-citations. Finally, one should also be aware that a country’s *h-*index depends on the citation database that is used [43].

Arab authors collaborated most with authors from Europe region, especially from France and Germany, followed with authors in the Asia-Pacific region, especially from India and Pakistan. This may be because the majority Arab researchers graduated from or trained in these countries. Furthermore, recently, many PhD students from the Arab world pursued their graduate ICM education in Europe and Asia-Pacific region, where the concept of ICM is being emphasized at the research and academic levels. Research collaboration is a significant way to improve quantity and quality of research at the university level [23]. Studies have found that there is a positive correlation between research output, and international or national collaboration at the researcher level [23, 44–46]. Previous studies have confirmed the significance of international collaboration in the quality of the research, which has a positive effect on citation rates [47–49]. A more recent study published in *the Lancet* to improve medical research in the Arab world recommended that an Arab medical research council-inspired from the US National Institutes of Health, the Medical Research Council in the United Kingdome, and French Institute of Health and Medical Research (INSERM) in France-is necessary to establish strategies that promote medical and health research in the Arab world in collaboration with international institutions [31].

Institutions of higher education in Arab region predominated in the most top 20 prolific institutions for research activity in ICM field; this indicates that institutions of higher education were actively researching and interesting in the ICM research. This may be attributed to universities encouragement for academics and researchers to publish their works in journals indexed in ISI database with impact factors [8, 20, 50]. Bibliometric measures is helpful in most countries to evaluate the quality and prestige of research centers and authors [51]. Research administrators use objective criteria for evaluating the performance of staff, departments and institutions-indeed entire countries. The alternative evaluation method is subjective depends on peer reviews by committees that undoubtedly has strong biases. Furthermore, using citation analysis for evaluation is not an ideal method, but after so many years it has got a level of standardization that permits one to obtain informed visions of their performance. Thus, the impact factors with the number of citations can achieve a better picture of performance. However, the journals’ impact factors become more important when evaluating a researcher’s most recent papers that have not yet been cited. In addition, the basic assumption is that if a document is published in a high impact journal, it most likely means something about the general quality of this paper. However, it is not a guarantee that it will be highly cited [51, 52].

As in any bibliometric study [8, 17, 22], the current study is not without limitations. First of all, we used Web of Science criteria for including ICM journals. Articles published in non-Web of Science-cited journals were not included; which might contribute to scientific research output. However, the 22 journals included in this study were the major international journals dedicated to the discipline of ICM. In addition, we searched only for journals included in the ICM category of JCR, however some articles in the ICM field may be published in other journals concerning ICM, with a wider field of interest, such as medicine and pharmacology. There are several databases that can be used to analyze scientific literature. Each database has its advantages and disadvantages [53–56]. Such comprehensive comparison among Web of Science, PubMed, and Scopus was discussed by different researchers [53–56]. For the purpose of this study, Web of Science was used because it offers several advantages over PubMed and Scopus with regard to the objective of the current study. First, in Web of Science various scientific disciplines are grouped into categories based on the scope of indexed journals. For example, in Web of Science, the category “Integrative & Complementary Medicine” allows researchers to retrieve documents published in ICM category. Second, Web of Science offers a powerful analysis of data from various aspects and all data can be easily transferred to Microsoft Excel for statistical analysis or graphics. A limitation of the method of ‘citation patterns’ is that older journals or articles are more likely to have been cited more, simply due to being around longer. Another limitation is that some publications from Palestine may be affiliated with Israel because SCI do not identify Palestine as a separate country. Therefore, some publications from Palestine might be not included in our analysis.

## Conclusions

Scientific research output in the ICM field in the Arab world region is increasing. Most of publications from Arab world in ICM filed were driven by societal use of medicinal plants and herbs. Search for new therapies from available low cost medicinal plants in Arab world has motivated many researchers in academia and pharmaceutical industry. Further investigation is required to support these findings in a wider journal as well as to improve research output in the field of ICM from Arab world region by investing in more national and international collaborative research project.

## Abbreviations

AI: Adjustment index
CAM: Complementary and Alternative Medicine
GDP: Gross domestic product
ICM: Integrative and Complementary Medicine
ISI: Institute for Scientific Information
IFs: Impact factors
JCR: Journal citation report
KSA: Kingdom of Saudi Arabia
SCR: Standard competition ranking
SPSS: Statistical package for social sciences
SAR: Syrian Arab Republic
USA: United States of America
UAE: United Arab Emirates
